# INCARCERATION HISTORY AND ITS ASSOCIATION WITH COGNITION AND ISOLATION AMONG OLDER BLACK AMERICANS

**DOI:** 10.1093/geroni/igad104.0873

**Published:** 2023-12-21

**Authors:** Eskira Kahsay, Rodlescia Sneed, Chuwen Zhong, Briana Mezuk

**Affiliations:** University of Michigan, Ann Arbor, Michigan, United States; Wayne State University, Detroit, Michigan, United States; School of Public Health, University of Michigan- Ann Arbor, Ann Arbor, Michigan, United States; University of Michigan, Ann Arbor, Michigan, United States

## Abstract

Incarceration is a risk factor for adverse health and disproportionately affects Black Americans. Little is known about its implications for later life functioning. This study investigated the associations between incarceration and social isolation and cognition among older Black Americans. Sample included African American and Caribbean Black respondents aged 50+ from the National Survey of American Life, 2001-2003 (N=1,561; 12.6% with incarceration history). Lifetime incarceration history (yes/no) was defined as having been in jail, prison, or juvenile detention. Continuous cognitive score was measured using the WHO Disability Assessment Schedule-II, with higher scores indicating higher cognitive difficulty. Binary isolation was assessed by combining two indicators of frequency of contact and feelings of closeness, for friends and family, using a 4-point Likert scale. Multivariable weighted linear and logistic regressions were fit for the associations between cognitive score and social isolation, respectively. All models adjusted for age, sex, ethnicity, poverty, education, marital status, and drug use; model for cognition further adjusted for mental health and traumatic experiences and models for isolation further adjusted for discrimination. Moderation by nativity (U.S. versus foreign born) was assessed using interaction. History of incarceration was associated with worse cognition (β = 0.09, 95% CI: -1.48, 1.66) and higher odds of social isolation for frequency (AOR: 1.48 95% CI: 0.89, 2.46) and closeness (AOR: 1.05, 95% CI: 0.52, 2.15). Results not moderated by nativity. Findings suggest contexts surrounding incarceration, like social supports, may be important engagement points for successful transitions and promotion of psychological and cognitive health in this population.

